# Early Elevation of Matrix Metalloproteinase-8 and -9 in Pediatric ARDS Is Associated with an Increased Risk of Prolonged Mechanical Ventilation

**DOI:** 10.1371/journal.pone.0022596

**Published:** 2011-08-03

**Authors:** Michele Y. F. Kong, Yao Li, Robert Oster, Amit Gaggar, J. P. Clancy

**Affiliations:** 1 Department of Pediatrics, University of Alabama at Birmingham, Birmingham, Alabama, United States of America; 2 Department of Medicine, University of Alabama at Birmingham, Birmingham, Alabama, United States of America; 3 Cincinnati Children's Hospital Medical Center, Cincinnati, Ohio, United States of America; Ludwig-Maximilians-Universität München, Germany

## Abstract

**Background:**

Matrix metalloproteinases (MMP) -8 and -9 may play key roles in the modulation of neutrophilic lung inflammation seen in pediatric Acute Respiratory Distress Syndrome (ARDS). We aimed to perform a comprehensive analysis of MMP-8 and MMP-9 activity in tracheal aspirates of pediatric ARDS patients compared with non-ARDS controls, testing whether increased MMP-8 and -9 activities were associated with clinical outcomes.

**Methods:**

Tracheal aspirates were collected from 33 pediatric ARDS patients and 21 non-ARDS controls at 48 hours of intubation, and serially for those who remained intubated greater than five days. MMPs, tissue inhibitor of metalloproteinases (TIMPs), human neutrophil elastase (HNE) and myeloperoxidase (MPO) activity were measured by ELISA, and correlated with clinical indicators of disease severity such as PRISM (Pediatric Risk of Mortality) scores, oxygen index (OI), multi-organ system failure (MOSF) and clinical outcome measures including length of intubation, ventilator-free days (VFDs) and mortality in the Pediatric Intensive Care Unit (PICU).

**Results:**

Active MMP-9 was elevated early in pediatric ARDS subjects compared to non-ARDS controls. Higher MMP-8 and active MMP-9 levels at 48 hours correlated with a longer course of mechanical ventilation (r = 0.41, p = 0.018 and r = 0.75, p<0.001; respectively) and fewer number of VFDs (r = −0.43, p = 0.013 and r = −0.76, p<0.001; respectively), independent of age, gender and severity of illness. Patients with the highest number of ventilator days had the highest levels of active MMP-9. MMP-9 and to a lesser extent MMP-8 activities in tracheal aspirates from ARDS subjects were sensitive to blockade by small molecule inhibitors.

**Conclusions:**

Higher MMP-8 and active MMP-9 levels at 48 hours of disease onset are associated with a longer duration of mechanical ventilation and fewer ventilator-free days among pediatric patients with ARDS. Together, these results identify early biomarkers predictive of disease course and potential therapeutic targets for this life threatening disease.

## Introduction

Acute Lung Injury (ALI), and in its most severe form, Acute Respiratory Distress Syndrome (ARDS) is a common, life-threatening cause of respiratory failure in children. Recent studies support a role for neutrophil-derived MMP-8 and -9, and an imbalance with their natural inhibitors, TIMPs in the pathogenesis of adult ALI and ARDS [1]–[5]. Despite our increasing understanding of the biologic activities of MMP-8 and-9, the role of these MMPs in pediatric ALI and ARDS remains ill defined. Examination of MMP activity in pediatric ARDS is important and potentially different than what has been described in adult ARDS for a number of reasons, including different etiologies underlying pediatric ARDS compared with adults, significant differences in immune function, and the potential for greater tissue plasticity during recovery from pediatric disease [6]. We previously identified high levels of MMP-8 and -9 relative to other MMPs in pooled lung secretions of children with ALI [7]. In this present study, we extend our previous observations by testing the hypothesis that MMP-8 and -9 are dysregulated early in the disease and have prognostic value in children with ARDS.

## Materials and Methods

### Ethic statement

This study was performed in the PICU at The Children's Hospital of Alabama with approval from the Institutional Review Board at the University of Alabama at Birmingham and written informed consent provided by legal guardians of the subject.

### Patient populations

ARDS subjects were children (≤17 years of age) who required mechanical ventilation and met clinical criteria for ARDS, including hypoxemia as defined by partial pressure of oxygen in arterial blood (PaO2)/fraction of oxygen in inspired air (FiO2) ≤200 mm Hg, bilateral infiltrates on chest radiograph and no clinical evidence of left atrial hypertension [8]. Patients were excluded if they had recent use of immunosuppresion including steroids or cytotoxic therapy. Control subjects were children (≤17 years of age) who required mechanical ventilation for non-pulmonary reasons, such as airway protection for head trauma or status epilepticus. All controls had no evidence of acute or chronic lung disease, with clear chest radiographs and minimal ventilatory requirement {low FiO2-ranging from 0.25-0.35, positive end expiratory pressure (PEEP) and ventilatory rate}. All radiographic interpretations were confirmed by independent readings from pediatric radiologists who were blinded to the study. Tracheal aspirates from study subjects were collected via endotracheal tube suctioning (with an 8F suction cannula, inserted past the endotracheal tube) within 48 hours of intubation.

### Data collection

Clinical data collected included patient demographics, diagnosis associated with the onset of ARDS, radiographic findings, arterial blood gases, OI, PaO_2_/FiO_2_ ratios, blood and tracheal aspirate culture data, PRISM scores, MOSF [9] and duration of mechanical ventilation. Number of ventilator-free days was defined as the number of days after the onset of ARDS per 28 day month [10] or intubation (for control subjects) that the patient was alive and not mechanically ventilated. During clinical data collection, the responsible research coordinator was blinded to all MMP and other biomarker data.

### Endotracheal aspirate processing

Endotracheal tube aspirates were collected on ice and centrifuged at 1000 RPM for 10 minutes, with separation of pellet from supernatant. Once the supernatant was collected, protein concentration was measured (Catalog # 5000112; Bio-Rad), and then separate aliquots were saved at 4°C for quantitative analysis of MMP-8, MMP-9, TIMP-1, MPO and HNE.

### Zymography

Zymography was performed on tracheal aspirates using a modified technique [11] to measure total gelatinase activity. In brief, samples (10 ug protein/sample) were subjected to electrophoresis through 7.5% polyacrylamide gels containing 1 mg/ml porcine skin gelatin in the presence of SDS under non-reducing conditions. Subsequently, gels were washed in 2.5% Triton X-100 for 1 hour at 4°C, then incubated in 50 mM TRIS and 5 mM CaCl for 16 hours at 37°C. Finally, gels were stained for 30 minutes in Coomassie blue and destained for 1–2 hours. MMP-9 activity present in samples was visible as lytic areas within the substrate gels.

### Measurement of active and total MMP-9 concentrations

MMP-9 activity was quantified using a fluorometric assay (R&D Systems # F9M00). 1 mM of Aminophenylmercuric acetate (APMA), a chemical activator of MMP-9 was added to selected samples to determine the total concentration of MMP-9 present, including pro-MMP-9. Complementary samples without the addition of APMA measured the amount of endogenously active MMP-9 present in the samples.

### Measurement of total MMP-8, TIMP-1, MPO and HNE concentrations

MMP-8, TIMP-1(R&D Systems # DMP800 and DTM100; respectively), MPO and HNE concentrations (Calbiochem # 475919 and CBA016; respectively) were measured using sandwich enzyme immunoassay techniques. MMP-8 activity was not routinely measured due to variable availability of standardized MMP-8 activity ELISA kits.

### Treatment of ARDS lower airway samples with MMP-8 and -9 inhibitors

Tracheal aspirates from ARDS subjects with high MMP-8 and -9 activity were incubated with EDTA (5 mM), MMP-8 specific inhibitor (Calbiochem #44237 at 30 ng/ml), MMP-9 specific inhibitor (Calbiochem #444278 at 50 ng/ml) and doxycyline (100 mcg/ml) or vehicle (DMSO, 1∶1000) for 2 hours followed by MMP-8 and -9 activity measurement (R&D Systems).

### Statistical analysis

Descriptive statistics were computed for each study variable of interest, including means, medians, standard error of the means (SEM), and ranges. Since the distributions of the data from the tracheal aspirate inflammatory markers deviated from a normal distribution, these data were logarithmically transformed using a log_10_ scale prior to statistical analysis. The log-transformed variables were determined to follow a normal distribution through the use of the Kolmogorov-Smirnov test and normal probability plots. Demographic and diagnostic comparisons between ARDS and control subjects were performed using the unpaired t-test for quantitative variables and the two-group chi-square test (or Fisher's exact test as needed) for categorical variables. Overall comparisons between ARDS and control subjects were performed using the unpaired t-test, while comparisons including covariates of interest (two disease etiologies, three disease severities, age, and gender) were performed using analysis of covariance. Comparisons between 48 hour and Day 6 measurements of total MMP-8, MMP-9 and active MMP-9 were performed using the paired t-test. Pearson correlation analysis was used to determine correlations between pairs of quantitative variables and Spearman correlation analysis was used to determine correlations between categorical variables (MOSF and gender) and quantitative variables. Simple and multiple linear regression analyses were used to assess the ability of the markers (active MMP-9, total MMP-9, percent active MMP-9, MMP-8, TIMP-1, MMP-9: TIMP-1, HNE, and MPO) to predict the outcome measures (days of intubation and ventilator-free days). Covariates included in the multiple regression analyses were age, gender, disease etiologies, P/F ratio, OI index, PRISM scores and MOSF. One-way analysis of variance was used to perform comparisons between the four quartiles for ARDS subjects. The Tukey-Kramer multiple comparisons test was then used to determine which specific pairs of means were significantly different. Where possible, nonparametric statistical analyses (including the Wilcoxon rank-sum test, the Wilcoxon signed rank test, the Kruskal-Wallis test, and Spearman correlation analyses) corresponding to the above analyses were performed, and these analyses yielded results that are similar to those obtained by the parametric analyses described above. Statistical tests were two-sided and were performed using a 5% significance level (i.e. alpha  = 0.05). Statistical analyses were performed with the use of SAS software (version 9.2; SAS Institute, Inc., Cary, NC).

## Results

A summary of demographic and diagnostic information regarding the pediatric ARDS and control subjects is shown in Table 1. 21 control subjects and 33 patients who met criteria for ARDS were enrolled prospectively over a two year period. In comparison to control subjects, ARDS patients had worse overall morbidity as indicated by longer duration of mechanical ventilation, fewer number of VFDs and higher PRISM scores on admission to the PICU (Table 1). At 48 hours of intubation and ARDS onset, the mean PaO_2_/FiO_2_ ratios and OI in the ARDS subjects was 153±12 and 18±5, respectively. ARDS was caused by a pulmonary etiology in 82% of all cases and 78% with a primary pulmonary process had an infectious etiology. Viral pneumonia, defined by a positive rapid viral screen or viral culture was seen in 41% {*influenza* (n = 5), *adenovirus* (n = 2), *parainfluenza* (n = 2) and *respiratory syncytial virus* (n = 2)}, while bacterial pneumonia, defined by a positive tracheal aspirate culture, was seen in 30% {*staphylococcus aureus* (n = 4), *pseudomonas aeruginosa* (n = 2), *haemophilus non-influenza* (1) and *streptococcus pneumoniae* (1)} of this cohort. None of the control subjects had a positive tracheal aspirate culture (viral or bacterial pathogens). Thirty-six percent of ARDS subjects had multi-organ failure and one succumbed to the disease. There were no fatalities in the control group, and none had multi-organ failure.

**Table 1:** Demographic and diagnostic information of ARDS and control subjects.

	Control	ARDS	P
**Number**	21	33	
**Age: Mean ± SEM** **(Range)**	6.5±1.4(4 weeks - 17 years)	6.6±1.1(6 weeks - 17 years)	0.95
**Gender: F:M**	6∶15	14∶19	0.30
**Length of intubation** **Mean days ± SEM (Range)**	4.3±0.6 (1 day - 10 days)	11±1.3 (4 days – 33 days)	<0.001
**Ventilator-Free Days** **Mean days ± SEM (Range)**	23.7±0.6(18 days – 27 days)	17.2±1.2(0 days – 24 days)	<0.001
* **PRISM 12 (Mean ± SEM)**	7.7±1.6	12.5±1.2	0.018
**PRISM 24 (Mean ± SEM)**	5.7±1.2	10.2±0.8	0.003
**†MOSF**	0	13 (39%)	<0.001
**Death**	0	1 (3%)	1.0
**Infectious**	0	20 (61%)	<0.001
**Pulmonary**	0	27 (82%)	<0.001
**Diagnosis**	Closed head injury (9)Seizure (3)Post laryngotrachealreconstruction (3)‡Others (6)	Bacterial pneumonia (8)Viral pneumonia (11)Aspiration (2)Pulmonary hemorrhage (2)Sepsis (6)Candidal pneumonia (1)Eosinophilic pneumonia (1)Smoke inhalation (1)Pulmonary contusion (1)	

*Pediatric Risk of Mortality; ^†^ Multi-organ System Failure; ^‡^ventricular shunt failure, post spinal fusion, choking, arrhythmia, spinal muscular atrophy, esophagitis.

### MMP-9, MMP-8, MPO and HNE profile in early ARDS

**Figure 1:**
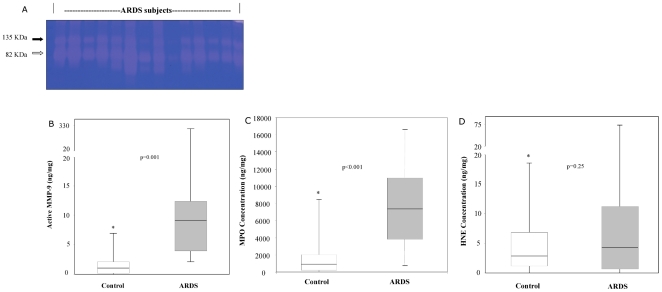
MMP-9, MPO and HNE levels in early pediatric ARDS. A. Zymogram (7.5% SDS non-reduced gel) demonstrates robust gelatinase activity in tracheal aspirates from ARDS subjects. Each lane represents a sample obtained from separate ARDS subjects at 48 hours of disease onset and intubation. The higher molecular weight band at 135 kDa (black arrow) likely represents lipocalin:MMP-9 complexes, while the 82 KDa band (white arrow) corresponds with active MMP-9 isoforms. B. Endogenously active MMP-9 levels measured in tracheal aspirates of ARDS subjects at 48 hours of disease onset and intubation were elevated (16-fold) compared with control subjects (*p = 0.001). Boxes represent 25^th^ to 75^th^ percentile range, with the midline indicating the median; bars represent the highest and lowest values. C. MPO concentrations were elevated (7-fold) in ARDS subjects compared to controls at 48 hours of intubation (*p<0.001). Boxes represent 25^th^ to 75^th^ percentile range, with the midline indicating the median; bars represent the highest and lowest values. D. HNE concentrations were elevated (3-fold) in ARDS subjects compared to controls at 48 hours of intubation (*p = 0.25). Boxes represent 25^th^ to 75^th^ percentile range, with the midline indicating the median; bars represent the highest and lowest values.

Tracheal aspirates from ARDS subjects were initially probed for MMP-9 activity by zymography. Figure 1A is an example of robust gelatinase activity found in ARDS subjects at 48 hours of intubation and ARDS diagnosis. In contrast, only sporadic lytic activity was seen in control subjects (not shown). Using ELISA techniques, the concentrations of endogenously active MMP-9 (i.e. prior to APMA stimulation) in tracheal aspirates from patients with ARDS were nearly 16-fold higher than controls (ARDS mean±SEM = 22.4±10 ng/mg vs. controls = 1.4±0.4 ng/mg; *p = 0.001*). Figure 1B demonstrates the range of this data, with the highest and lowest values, and median data for both the ARDS and control group. Using multivariate analysis to adjust for differences in disease etiology (pulmonary versus non-pulmonary, infectious versus non-infectious) and disease severity (PRISM scores and MOSF) between ARDS and non-ARDS subjects, up-regulation of active MMP-9 seen in the ARDS cohort remained statistically significant (*p = 0.002*). Comparison of total MMP-9 concentrations (includes both basal activity and inducible pro-enzyme) between the ARDS subjects and controls also demonstrated statistically significant differences between the groups (measured after APMA stimulation to assess pro-MMP-9 concentrations; ARDS mean±SEM = 184±36 ng/mg vs. controls = 95±26 ng/mg; *p = 0.009*). Likewise, total MMP-8 concentrations were higher in the ARDS subjects compared to non-ARDS controls (ARDS mean±SEM = 951±242 ng/mg vs. control = 384±97 ng/mg; *p = 0.043*). However, no differences were observed for total MMP-8 and -9 concentrations between ARDS and control subjects after adjusting for disease etiology and severity of illness.

As surrogates of pulmonary neutrophil influx, MPO and HNE concentrations in tracheal aspirates were measured in all subjects. MPO levels from ARDS patients were found to be approximately 7-fold higher than in controls (MPO in ARDS mean±SEM = 9262±1696 ng/mg vs. control = 1396±439 ng/mg; *p<0.001*; Figure 1C) which was confirmed by analysis of covariance (*p = 0.004*). For HNE levels, differences were present between ARDS and control subjects (HNE in ARDS mean±SEM = 12.5±4 ng/mg vs. control = 4.4±1.1 ng/mg; *p = 0.25*; Figure 1D) but did not meet statistical significance by univariate and multivariate analysis. MPO and HNE correlated positively with each other (Table 2; r = 0.63; *p<0.001*) and both MMP-8 and MMP-9 levels demonstrated positive correlations with MPO and HNE concentrations (Table 2), consistent with common sources for these enzymes. The correlations ranged from moderate (MMP-9 with HNE and MPO, MMP-8 with HNE and MPO) to low (MMP-8 and MMP-9).

**Table 2:** Correlation coefficients for tracheal aspirate inflammatory markers at 48 hours of ARDS.

	r	P
**HNE and MPO**	0.63	<0.001
**Total MMP-9 and HNE**	0.47	0.006
**Total MMP-9 and MPO**	0.46	0.007
**MMP-8 and HNE**	0.56	<0.001
**MMP-8 and MPO**	0.67	<0.001
**Total MMP-8 and MMP-9**	0.38	0.031

Note: All correlations are indicative of positive relationships between these pairs of variables.

### TIMP-1 and MMP-9:TIMP-1 activity ratios in ARDS

TIMP-1 concentrations were slightly lower in the ARDS subjects compared to controls (mean±SEM = 113±15 ng/mg vs.144±27 ng/mg, respectively; p = 0.43), contributing to higher MMP-9:TIMP-1 activity ratios in ARDS subjects compared to controls (mean±SEM = 2.7±0.6 vs. 1.4±0.5, respectively; *p = 0.039*).

### Higher active MMP-9, fraction of active MMP-9 and total MMP-8 levels at 48 hours predicts longer ARDS-related ventilator support

At 48 hours of intubation and ARDS onset, increasing active MMP-9 levels and fraction of active MMP-9 (active MMP-9/total MMP-9) had strong positive correlation (r = 0.75; *p<0.001* and 0.61; *p<0.001*, respectively) with the need for prolonged mechanical ventilation in the ARDS subjects. Similarly, higher total MMP-8 levels at 48 hours of intubation correlated with subjects who had longer ARDS-related ventilator support (r = 0.41; *p = 0.018*). ARDS patients with higher active MMP-9 and fraction of active MMP-9 at 48 hours of disease onset also had lower number of VFDs (r = −0.76; *p<0.001* and −0.60; *p<0.001,* respectively). ARDS subjects with higher total MMP-8 concentrations at 48 hours also had lower number of VFDs (r = −0.43; *p = 0.013*). Multiple regression analyses confirmed the observation that higher levels of active MMP-9, percent active MMP-9 and total MMP-8 at 48 hours of ARDS onset predicted the duration of mechanical ventilation (*p<0.001*, *p = 0.005* and *p = 0.004*; respectively), after adjusting for disease etiology (pulmonary vs. non-pulmonary, infectious vs. non-infectious), disease severity (ex: PRISM scores, presence of MOSF), oxygenation defect (ex: P/F ratios, OI), age and gender. Similarly, multiple regression analyses demonstrated that higher active MMP-9, percent active MMP-9 and total MMP-8 correlated with fewer number of VFDs in the ARDS subjects (*p<0.001*, *p = 0.004* and *p = 0.004*; respectively).

No significant correlations were identified between total MMP-9, TIMP-1, MMP-9:TIMP-1 ratios, HNE or MPO with duration of mechanical ventilation and VFDs in the ARDS subjects. Similarly, no clear relationship was observed between the measured biomarkers and clinical indicators of disease severity (P/F ratios, OI, PRISM 12, PRISM 24 and MOSF). In the control non-ARDS group, no correlation was observed between the tracheal aspirate biomarkers (active MMP-9, total MMP-9, percent active MMP-9, MMP-8, TIMP-1, MMP-9:TIMP-1, HNE and MPO) and clinical markers of disease severity and outcome measures (P/F ratios, OI, PRISM 12, PRISM 24, MOSF, duration of intubation and VFDs).

To further examine relationships between duration of intubation and measured MMP-8 and MMP-9 levels at 48 hours of ARDS, we stratified ARDS subjects into quartiles based on number of ventilator days (Table 3). The mean duration of mechanical ventilation in the top quartile was 21.9 days, strikingly longer than the bottom 3 quartiles (10.5±0.6, 7.7±0.4 and 4.8±0.2, respectively, *p<0.001*). Endogenously active MMP-9 concentrations measured at 48 hours of ARDS were highest in patients who went on to have the longest duration of mechanical ventilation (67.5±12 ng/mg vs. 7.4±1.2 ng/mg in those with the fewest ventilator days, *p<0.001*; Table 3). Total MMP-9 concentration was not significantly different across the 4 quartiles. When examined by ratio of active to total MMP-9, patients in the top quartile of ventilator days had the highest active fraction (53%±9), in contrast to MMP-9 detected predominantly in the latent form for patients in the 2^nd^, 3^rd^ and 4^th^ quartile of ventilator days (active fraction <15% across all three quartiles, *p<0.001*). In contrast to MMP-9 measurements, the MMP-8 levels trended towards a continuous increase across the quartiles of increasing duration of ventilatory support (*p = 0.08*). Measured TIMP-1, MMP-9:TIMP-1 ratios, MPO and HNE concentrations showed no clear relationships with duration of mechanical support.

**Table 3:** Examination of 48 hour inflammatory markers in ARDS subjects, segregated into quartiles for length of intubation.

	1st Quartile(n = 8)	2nd Quartile(n = 8)	3rd Quartile(n = 8)	4th Quartile(n = 9)	Overall p-value
**Duration of intubation (Days)**	21.9±2.9^a^	10.5±0.6^b^	7.7±0.4^b,c^	4.8±0.2^c,d^	<0.001
**Active MMP-9 (ng/mg)**	67.5±12.1^a^	9.7±2.5^b^	7.1±1.7^b^	7.4±1.2^b^	<0.001
**Total MMP-9** **(ng/mg)**	176±68	190±55	278±82	80±17	0.31
* **Fraction of** **Active MMP-9**	52.8±8.7^a^	14.5±7.3^b^	5.5±1.6^b^	11.5±2.0^b^	<0.001
**TIMP-1 (ng/mg)**	116±33	106±32	136±32	89±27	0.64
**MMP-9:TIMP-1 ratios**	3.3±1.4	3.9±2.0	2.3±0.6	1.4±0.3	0.79
**Total MMP-8 (ng/mg)**	1595±505	1227±476	729±261	281±82	0.087
**HNE (ng/mg)**	15.7±9.2	22.5±12.3	7.1±2.9	5.3±2.0	0.72
**MPO (ng/mg)**	11562±3646	11638±5581	7576±1672	6481±1215	0.62

*Active MMP-9/Total MMP-9 in %.

Data are presented as means ± SEM. Means in a row with different superscript letters are significantly different, p<0.05. Only active MMP-9 and fraction of active MMP-9/total MMP-9 demonstrated positive correlation with length of intubation (p<0.001), while total MMP-8 demonstrated a trend towards correlation with length of intubation.

### Rising levels of active MMP-9 are seen in protracted ARDS

**Figure 2:**
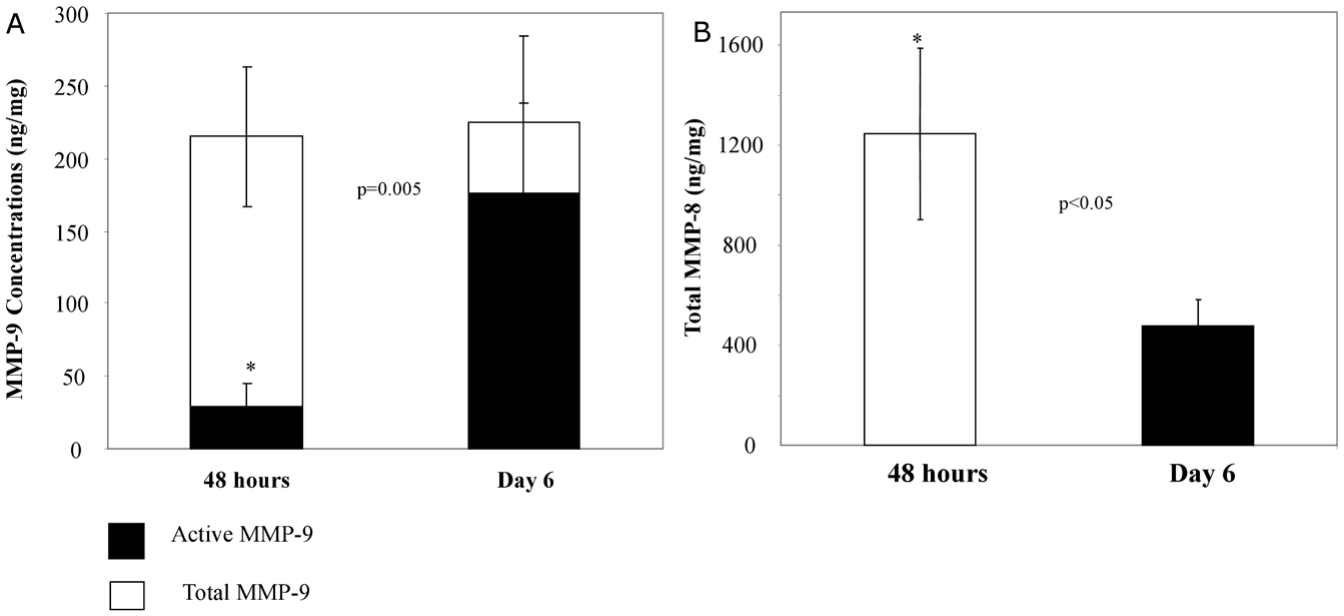
MMP-9 and MMP-8 levels in protracted ARDS. A: Increase in active MMP-9 with ARDS progression. Total MMP-9 remained elevated in ARDS patients intubated for >5 days (n = 25). With ARDS progression, approximately 80% of MMP-9 was found in the active form (prior to APMA stimulation). Active MMP-9 at Day 6 of ARDS was 6-fold higher than at 48 hours of disease (mean±SEM 176±61 ng/mg vs. 30±16 ng/mg at 48 hrs, respectively; *p = 0.005). B: Decline in MMP-8 levels with ARDS progression. Total MMP-8 decreased with ARDS progression in those who remained intubated for >5 days (n = 25, mean±SEM 1245±343 ng/mg at 48 hrs vs. 537±122 ng/mg at day 6, respectively; *p<0.05).

Due to consistent covariate analysis demonstrating a significant relationship between MMP-9 activity and the length of mechanical ventilation, we analysed MMP-9 activity profile with ARDS progression. Of the 33 ARDS subjects, 25 went on to require more than five days of mechanical ventilation and continued to meet the diagnostic criteria for ARDS. In this group of patients, a second sample was obtained between 5–10 days of intubation (mean±SEM = 7.1±0.3 days, median = 6 days). The concentrations of active MMP-9 in these second samples were nearly 6-fold higher than at 48 hours (Figure 2A; *p = 0.005*). Unlike the first sample obtained 48 hours after intubation, most of the MMP-9 measured in the 2^nd^ sample was active (80% vs. 14%, respectively; *p = 0.005*; Figure 2A). Total MMP-8 levels in ARDS patients demonstrated a very different pattern over time with a two-fold reduction seen at day 6 (*p = 0.05*; Figure 2B). Parallel studies performed in non-ARDS controls who were intubated for greater than 5 days (n = 5; mean±SEM = 7±0.7 days) demonstrated minimal changes in MMP-8 and MMP-9 activity with time on the ventilator. In contrast to ARDS subjects, less than 1% of MMP-9 was found in the active fraction in these control samples collected between days 5-10 of intubation (vs. 80% in ARDS).

### Reduction of excessive MMP-8 and MMP-9 in ARDS tracheal aspirates by MMP inhibitors

**Figure 3:**
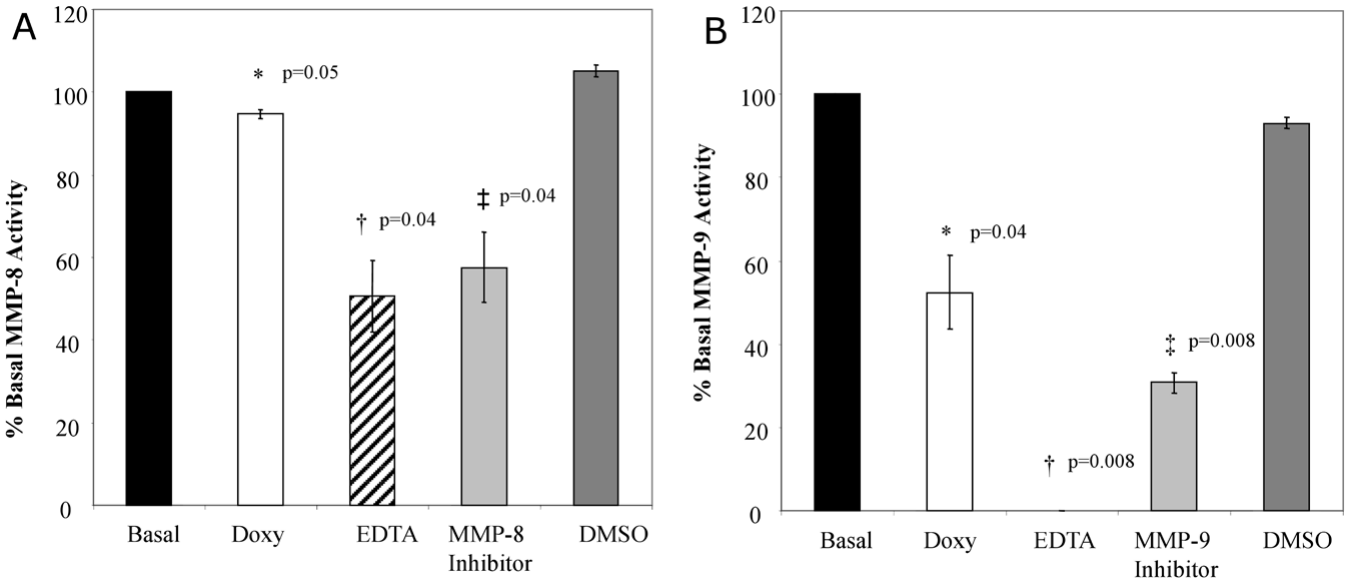
Treatment of ARDS tracheal aspirates with MMP-8 and MMP-9 inhibitors. A. Tracheal aspirates from ARDS subjects (n = 4) with high MMP-8 activity were examined in the presence of different MMP inhibitors. MMP-8 was reduced by 40–50% relative to basal activity when incubated with 5 mM of EDTA (^†^ *p = 0.04*) and 30 ng/ml of a specific MMP-8 inhibitor (^‡^ *p = 0.04*) versus basal activity and vehicle controls. Incubation with 100 mcg/ml of doxycycline resulted in a 10% decrease in MMP-8 activity compared to basal levels (*p = 0.05). B. Tracheal aspirates from ARDS subjects (n = 4) with high MMP-9 activity were examined in the presence of different MMP inhibitors. MMP-9 activity decreased by 70–100% relative to basal activity in the presence of EDTA (5 mM; ^†^ *p = 0.008*) and a MMP-9 specific inhibitor (50 ng/ml; ^‡^ *p = 0.008*). Inhibition with doxycycline (100 mcg/ml) decreased MMP-9 activity by approximately 40% of basal activity (*p = 0.04).

To determine if small molecule inhibitors of MMPs could normalize MMP-8 and MMP-9 activity in pediatric ARDS tracheal aspirates, samples from subjects with high MMP-8 and MMP-9 activity (n = 4) were treated with specific and nonspecific MMP inhibitors, and MMP activity was measured. MMP-8 activity was reduced by approximately 40% with EDTA and a specific MMP-8 inhibitor (Figure 3A; *p = 0.04*). Measurement of MMP-9 activity demonstrated an approximate 60–100% reduction in activity by EDTA and a specific MMP-9 inhibitor (Figure 3B; *p = 0.008*) relative to vehicle control. Treatment of ARDS samples with doxycycline, an antimicrobial agent with MMP inhibitory properties [12] resulted in a 50% reduction in MMP-9 activity (Figure 3B, *p = 0.04*). A less dramatic inhibition was seen with MMP-8, with only ≈10% reduction in activity produced by doxycycline (Figure 3A; *p = 0.05*).

## Discussion

ARDS is a syndrome of severe pulmonary dysfunction and refractory hypoxemia that results from a massive neutrophilic inflammatory response [13]. Previous reports have implicated MMP-8 and MMP-9 in the modulation of neutrophil mediated lung inflammation [14]–[18], and support the hypothesis that MMP-8 and -9 play key roles in neutrophil-mediated inflammation, which is central to the pathogenesis of ARDS.

In this study, we examined MMP-8 and MMP-9 levels in pediatric ARDS, exploring relationships between their concentrations, activity and modulation with disease progression. These two MMPs were chosen based on our previous work in which comprehensive MMP screening demonstrated that MMP-8 and 9 were among a limited number of MMPs found to be elevated in pediatric ALI [7]. In this study, we identified significant increases of active MMP-9 in a series of 33 children with ARDS compared to non-pulmonary disease controls at 48 hours of intubation, independent of age, gender, disease etiology and severity. In addition, the active fraction of MMP-9 further increased with ARDS progression. These findings point towards dysregulation of MMP-9 in ARDS, with depressed TIMP-1 levels contributing only in part to this effect. In control subjects, no significant changes were seen in MMP-8 and MMP-9 over time while subjects remained on the ventilator. Furthermore, almost all of the MMP-9 detected was inactive in our ventilator-control group, indicating the presence of normal MMP-9 regulation (not shown).

In the current report, both MMP-8 and -9 had positive correlations with MPO and HNE, enzymes present in abundance in neutrophil granules, suggesting that neutrophils were an important source of the MMPs. In comparison to MMP-8, MMP-9 had a weaker correlation with MPO and HNE. Since more than one-third of our ARDS subjects had viral induced ARDS, epithelia derived MMP-9 {described in respiratory viral infections [19]} may potentially have contributed to high MMP-9 levels, in addition to MMP-9 released from activated neutrophils.

To date, there have been contradictory reports regarding whether MMP-9 is protective or harmful in lung injury models [20]–[23]. In the current study, subjects who went on to require longer ARDS-related ventilator support and had fewer ventilator-free days demonstrated higher active MMP-9, fraction of active MMP-9 and total MMP-8 levels at 48 hours of ARDS, independent of age, gender, oxygenation defect, disease etiology and severity of illness. This observation suggests that up-regulation of these MMPs early in disease predicted a more protracted ARDS course. This was seen most dramatically in patients with very prolonged ventilator support (Table 3), who had extremely high levels of active MMP-9. The findings support the hypothesis that active MMP-9 may serve as a biomarker to predict the length of ventilator support in the pediatric ARDS population. The relationships between MMP levels and other clinical parameters such as PaO_2_/FiO_2_ ratios, OI, PRISM scores and MOSF were less clear than their relationships with duration of ventilator support. While we observed no correlation between MMP levels and ARDS related mortality, firm conclusions cannot be drawn as only one ARDS subject died from ARDS related complications. In this one ARDS subject, MMP-9 activity and MMP-9:TIMP-1 ratio remained elevated throughout his disease course.

Elevated MMP-8 and MMP -9 activity in ARDS lung secretions could be reduced by treatment with MMP inhibitory molecules ex vivo (Figure 3). Tetracycline derivatives have been demonstrated to be potent MMP inhibitors [12], [24]. In a study by Kim JH et al, MMP-9 inhibition by pre-treatment with a chemically modified tetracycline derivative decreased the degree of ventilator induced lung injury in rats exposed to high volume ventilation [20]. Other studies using acute animal models of lung injury such as after cardiopulmonary bypass [21] and after infusion of *Escherichia coli* lipopolysaccharide [25] also demonstrated that blocking of proteases, primarily MMP-9 and neutrophil elastase with a modified tetracycline (COL-3) prevented lung injury and ultimately the development of ARDS. COL-3 also completely prevented all the pathological lung changes seen in a porcine model of sepsis induced ARDS [26]. In our study, EDTA, MMP specific inhibitors and doxycyline inhibited MMP-9 and to a lesser extent MMP-8 activity, suggesting that strategies utilizing small molecule agents to restore MMP regulation may retain activity in the ARDS environment. These findings provide support for further studies examining cause-effect relationships between MMP activity and the pathogenesis of ARDS.

Patients who were on steroids or immunosuppressive medications prior to intubation (both for control and ARDS subjects) were excluded from the study secondary to the potential effect of these medications on MMP expression and activity. However, out of the 33 enrolled ARDS subjects, three received hydrocortisone (100 mg/m^2^/day) for shock and five received solumedrol (2 mg/kg/day) for wheezing within the first two days of intubation. No correlation was found between the use of steroids and MMP expression or with markers of disease severity and outcome measures. None of the control subjects received steroids during the course of their intubation. However, due to the small number of patients who received steroids in our cohort, firm conclusions cannot be drawn.

One limitation of this study is that it was performed in one tertiary pediatric medical institution only. Future examination of MMP-8 and -9 as biomarkers of pediatric ARDS would benefit from a larger multicenter approach. Although this study demonstrated up-regulation of active MMP-9 in pediatric ARDS subjects and association between increased MMP-8 and MMP-9 early in disease course with duration of mechanical ventilation, it is crucial to recognize that examination of MMP-8/9 levels with other previously recognized ARDS biomarkers such as plasma von Willebrand factor antigen, plasminogen activator inhibitor-1 and B-type natriuretic peptide [27]–[29] may provide greater prognostic value than MMP biomarkers alone. Finally, although we demonstrate robust blockade of MMP-8/9 activity in tracheal aspirates from ARDS subjects, it is unknown whether in-vivo blockade of these proteases may alter the disease course and outcome. However, this study provides the foundation to support potential therapeutic trials examining specific agents with MMP inhibitory activities (such as doxycyline) as modulators of excessive MMP-8 and/or MMP-9 activity in children with ARDS.

### Conclusion

The results of this study confirm our previous reports that MMP-8 and -9 are increased in pediatric ARDS, and provide evidence that early elevation of MMP-8 and MMP-9 levels, particularly active MMP-9 predicts the duration of mechanical ventilation. This relationship is independent of age, gender, disease etiology and disease severity. These findings support a role of protease dysregulation in the pathogenesis of pediatric ARDS, and suggest that elevated MMP-8 and/or active MMP-9 may have a prognostic value in pediatric ARDS patients. Larger prospective studies are warranted to confirm these findings with a goal of determining whether MMP-8/9 are appropriate targets to modify patient outcomes.
